# Consistent elicitation of cross-clade HIV-neutralizing responses achieved in guinea pigs after fusion peptide priming by repetitive envelope trimer boosting

**DOI:** 10.1371/journal.pone.0215163

**Published:** 2019-04-17

**Authors:** Cheng Cheng, Kai Xu, Rui Kong, Gwo-Yu Chuang, Angela R. Corrigan, Hui Geng, Kurt R. Hill, Alexander J. Jafari, Sijy O’Dell, Li Ou, Reda Rawi, Ariana P. Rowshan, Edward K. Sarfo, Mallika Sastry, Kevin O. Saunders, Stephen D. Schmidt, Shuishu Wang, Winston Wu, Baoshan Zhang, Nicole A. Doria-Rose, Barton F. Haynes, Diana G. Scorpio, Lawrence Shapiro, John R. Mascola, Peter D. Kwong

**Affiliations:** 1 Vaccine Research Center, National Institute of Allergy and Infectious Diseases, National Institutes of Health, Bethesda, Maryland, United States of America; 2 Duke Human Vaccine Institute, Duke School of Medicine, Durham, North Carolina, United States of America; 3 Department of Biochemistry and Molecular Biophysics, Columbia University, New York, New York, United States of America; Emory University School of Medicine, UNITED STATES

## Abstract

The vaccine elicitation of broadly neutralizing responses is a central goal of HIV research. Recently, we elicited cross-clade neutralizing responses against the N terminus of the fusion peptide (FP), a critical component of the HIV-entry machinery. While the consistency of the elicited cross-clade neutralizing responses was good in mice, it was poor in guinea pigs: after seven immunizations comprising either envelope (Env) trimer or FP coupled to a carrier, serum from only one of five animals could neutralize a majority of a cross-clade panel of 19 wild-type strains. Such a low response rate—only 20%—made increasing consistency an imperative. Here, we show that additional Env-trimer immunizations could boost broad FP-directed neutralizing responses in a majority of immunized animals. The first boost involved a heterologous Env trimer developed from the transmitted founder clade C strain of donor CH505, and the second boost involved a cocktail that combined the CH505 trimer with a trimer from the BG505 strain. After boosting, sera from three of five animals neutralized a majority of the 19-strain panel and serum from a fourth animal neutralized 8 strains. We demonstrate that cross-reactive serum neutralization targeted the FP by blocking neutralization with soluble fusion peptide. The FP competition revealed two categories of elicited responses: an autologous response to the BG505 strain of high potency (~10,000 ID_50_), which was not competed by soluble FP, and a heterologous response of lower potency, which was competed by soluble FP. While the autologous response could increase rapidly in response to Env-trimer boost, the heterologous neutralizing response increased more slowly. Overall, repetitive Env-trimer immunizations appeared to boost low titer FP-carrier primed responses to detectable levels, yielding cross-clade neutralization. The consistent trimer-boosted neutralizing responses described here add to accumulating evidence for the vaccine utility of the FP site of HIV vulnerability.

## Introduction

The induction of immune responses capable of neutralizing a substantial portion of circulating HIV-1 strains has been a major goal of vaccine research. Unlike influenza virus, where the majority of variation is found in animal reservoirs and the circulating diversity in humans is <1% of the viral genome, the circulating diversity in humans of HIV-1 is roughly 30% at the amino-acid level [1]. This level of diversity is much greater than the circulating diversity found with most other viruses; indeed, it is estimated that the diversity of HIV-1 in a single individual infected for six years is roughly equivalent to the annual diversity of circulating influenza sequences world-wide [2]. In addition to genetic variation, the trimeric envelope (Env) glycoprotein of HIV-1 uses conformational masking and an evolving glycan shield to evade neutralizing antibody [3, 4]. Together, these immune evasion mechanisms have confounded HIV vaccine efforts. While vaccine-induced neutralization has been achieved for autologous virus [5–8], where the neutralized strain is the same strain from which the immunogen was developed, vaccine-induced neutralization of heterologous strains has only been achieved sporadically in select animals [9, 10].

Recently, we described a vaccination scheme based on fusion peptide (FP), a critical element of the HIV-1 entry machinery. FP comprises a hydrophobic region (residues 512–527) at the N terminus of the gp41 transmembrane subunit of the HIV-1 Env trimer. Most of FP is occluded in the prefusion conformation of Env, before being embedded in the target cell membrane when gp41 forms an extended structure called the ‘prehairpin’ intermediate, wherein FP anchors Env in the target cell membrane to facilitate fusion of virus and cell membrane [11, 12]. We found that immunization with the N-terminal half of FP (residues 512–519; FP8) followed by Env trimer boosts could elicit cross-clade neutralizing responses in standard vaccine-test species, including mice, guinea pigs, and rhesus macaques [13]. In C57BL/6 mice, cross-clade neutralizing responses could be induced in a majority of animals by priming with FP8 coupled to keyhole limpet hemocyanin (KLH), a standard carrier protein for immunization, and boosting with prefusion-stabilized Env trimers. While use of these immunogens could also induce cross-clade neutralizing responses in guinea pigs and rhesus macaques, animals with serum neutralization breadth of 20% or higher—as assessed on a panel of 208-isolates—were only observed sporadically [13].

Here we test the ability of a heterologous Env trimer [9, 14] developed from the clade C-infected donor, CH505 [15–17], to increase the consistency of broad FP-directed responses. In specific, we assessed in guinea pigs already immunized seven times with FP coupled to KLH or with BG505 Env trimers the impact of two additional Env trimer boosts, the first a CH505 Env trimer and the second a cocktail of CH505 and BG505 Env trimers, on ELISA responses and on neutralization breadth as measured on a 19-strain panel. This panel comprised BG505 and two subpanels: one with 9 heterologous wild-type viruses with matching FP8 sequence (‘FP-matching’ subpanel, in which the FP8 sequence was AVGIGAVF, the most abundant FP8 sequence), and the other with 9 heterologous wild-type viruses with different FP8 sequences (‘FP-nonmatching’ subpanel). We also characterized the impact on neutralization of competition with FP peptide. Overall, we found Env trimer boosts to increase the consistency of cross-reactive FP-directed HIV-1-neutralizing responses by boosting what appeared to be previously primed FP responses to detectable levels.

## Results

### Env-trimer boosts achieve broad FP-directed responses in 3 of 5 animals

#### FP-matching subpanel

**Fig 1:**
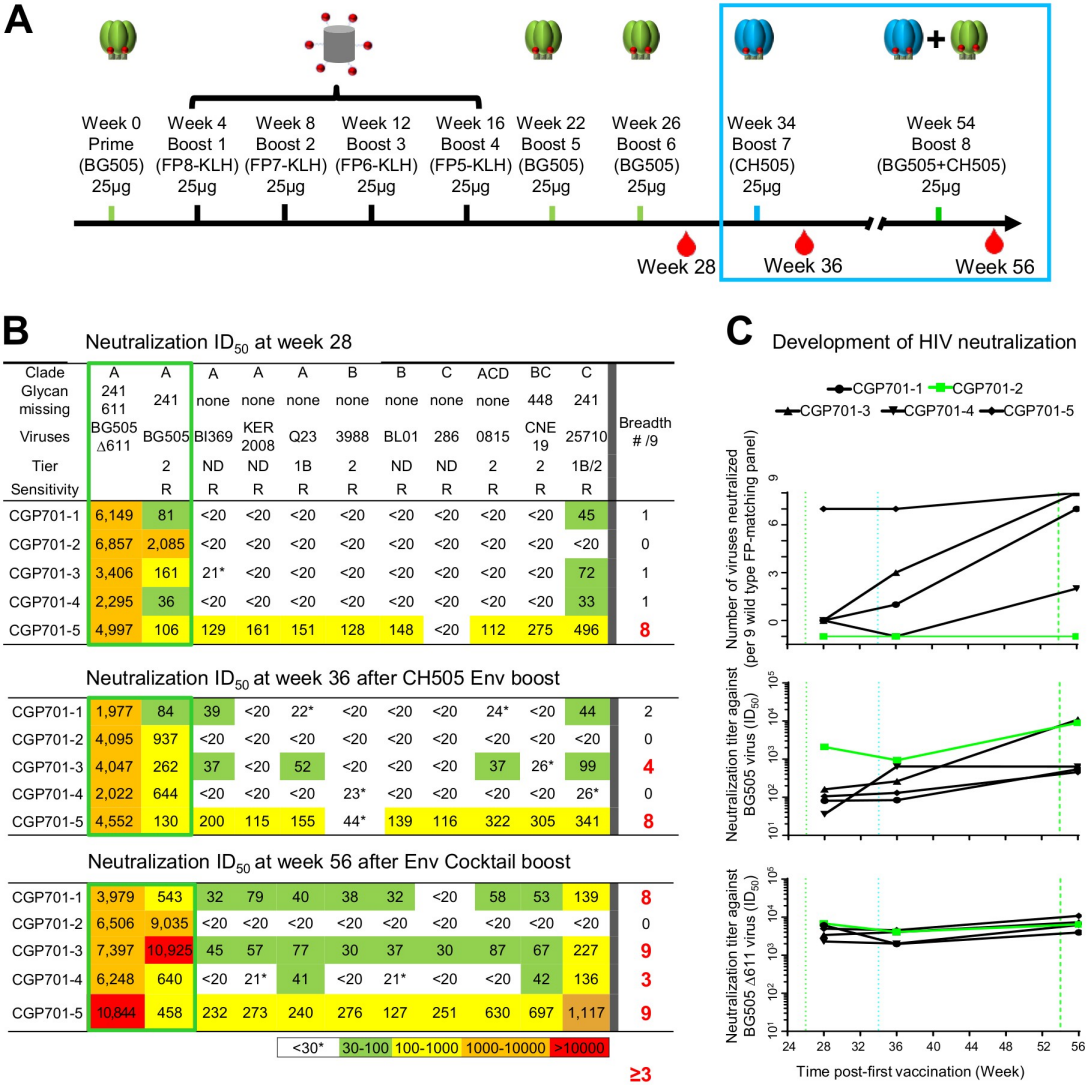
Additional Env trimer boosts increase cross-clade neutralizing responses in 4 of 5 guinea pigs against HIV strains with matching FP8 sequence. **A**. Immunization scheme. Initial seven immunizations described in Xu et al. [13], with final two boosts (blue outline) at weeks 34 and 54 described here. **B**. Serum neutralization ID_50_ titer of HIV-1 strains for autologous BG505 (wild-type and Δ611-mutant) (green box, left) and 9 FP-matching heterologous wild-type strains (middle) with FP-matching breadth (right) for week 28 (top), week 36 (middle), and week 56 (bottom). See S5 Table for assessments on non-HIV-1 viruses and S1 Fig for representative neutralization curves. *Neutralization titers were considered positive when ID_50_ ≥30 for CGP701-1 through CGP701-4 or when ID_50_ ≥50 for CGP701-5. HIV-1 strains are categorized as resistant (R) or sensitive (S) based on their neutralization by five antibodies: 17b, 48b, F105, 3074 and 447-52D, which generally neutralize only Tier 1 isolates. ND: Tier status not determined. **C**. Longitudinal development of HIV neutralization. Dashed vertical lines indicate the boosts at week 28, 34 and 54. CGP701-2 showed primarily autologous responses, and titers for this animal are highlighted in green.

We previously immunized a group of five guinea pigs with BG505 DS-SOSIP Env trimer followed by four immunizations with FP-KLH immunogens in which FP was 8-residues for the first immunization (FP8) and decreased by one C-terminal residue for each of the subsequent immunizations (FP7, FP6, and FP5) [13]. After an additional two boosts with BG505 DS-SOSIP Env trimer, all animals could neutralize the BG505 autologous virus by week 28 (Fig 1A and 1B). High titers (2000–7000) were observed against the Δ611 BG505 glycan variant, which is ~10-100-fold more sensitive to neutralization by FP-directed antibodies than wild-type [13, 18].

We assessed the ability of the guinea pig sera to neutralize a cross-clade panel of nine wild-type viruses, all with the most prevalent FP8 sequence, AVGIGAVF. We termed this panel the ‘FP-matching subpanel’. The nine strains of this panel comprised seven strains from a 10-strain panel we had used previously to assess FP-directed responses [13] and two new strains (BI369 from clade A and 0815 from clade ACD, both with matching FP8 sequence). Notably, all 9 strains on the matching panel were resistant to neutralization by antibodies 17b, 48d, F105, 3074, and 447-52D (which generally neutralize only laboratory-adapted strains) (S1 Table), though only three were formally designated as Tier 2, with another of Tier 1B/2 status, another of Tier 1B status, and four of unknown Tier (Fig 1B). Sera from only one animal, CGP701-5, could neutralize a majority of the nine strains, whereas the other four animals only neutralized 0 or 1 heterologous virus (Fig 1B, top panel).

We tested the impact of further immunizations using a soluble Env-trimer protein developed from the clade C CH505 donor in which the N and C termini of gp120 and gp41 derived from the clade A BG505 strain, while the rest was from CH505 [14]. Importantly, we chose this CH505 chimeric trimer because it displays the full complement of glycans around FP, including *N*241, which is absent in the BG505 trimer. Moreover, broadly neutralizing antibodies were identified from the CH505 donor [15–17] and use of this Env trimer elicited neutralizing responses in select rabbits and rhesus macaques [9]. After CH505-Env trimer immunization, we observed an increase in breadth, as assessed on the FP-matching subpanel, in two of five animals at week 36 (Fig 1B, middle panel).

At week 54, we further boosted with an Env-trimer cocktail containing a mixture of both CH505 and BG505. We observed neutralization breadth to increase further, with three of five animals now capable of neutralizing a majority of viruses on the FP-matching subpanel (Fig 1B, bottom panel). Indeed, only one animal, CGP701-2, failed to show heterologous neutralization on the FP-matching subpanel. Overall, while neutralization titers against BG505 or the Δ611 variant showed only small increases, heterologous breadth showed an increase in four of the five animals after additional Env-trimer boosts (Fig 1C, top panel).

#### FP-nonmatching subpanel

**Fig 2:**
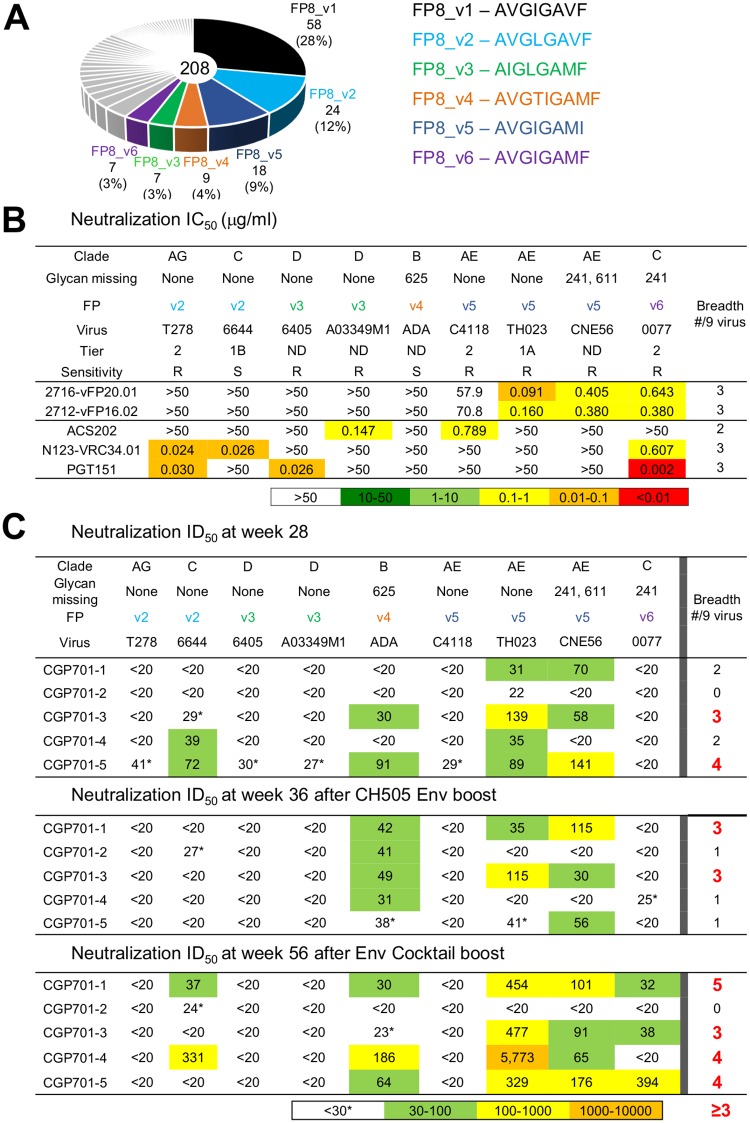
Additional Env trimer boosts increase neutralization titer against HIV strains with non-matching FP8 sequences. **A**. FP8 sequence diversity among 208 Env sequences. **B**. FP-directed antibody neutralization of viruses with FP non-matching sequences. HIV-1 strains were categorized as resistant (R) or sensitive (S) based on neutralization by five HIV-1 neutralizing antibodies:17b, 48b, F105, 3074 or 447-52D. Tier sensitivity is indicated when known. **C**. Serum neutralization of viruses with FP non-matching sequences, for week 28 (top), week 36 (middle), and week 56 (bottom). *Neutralization titers were considered positive when ID_50_ ≥30 for CGP701-1 through CGP701-4 or when ID_50_ ≥50 for CGP701-5 (see S5 Table for assessments on non-HIV-1 viruses).

We next focused on the ability of the sera to recognize different FP8 sequences (Fig 2). We analyzed the prevalence of FP8 sequences, and chose representative viruses which had FP8 sequences corresponding to the five next most prevalent sequences (FP8_v2 through FP8_v6) on our 208-strain panel [19] (Fig 2A). For the non-matching panel, we chose primarily neutralization resistant strains, though we also chose two strains, 6644.V2.C33 and ADA.DG, which were sensitive to neutralization by antibodies F105, 3074 or 447-52D.

The vaccine-elicited murine antibodies, vFP16.02 and vFP20.01, which we described previously [13] could neutralize three of the nine selected strains on this FP-nonmatching subpanel, tolerating variation at the C-terminal region of the FP8 sequence, but not of the first five residues of FP. Meanwhile, the naturally elicited antibodies PGT151, VRC34.01 and ACS202 each neutralized either two or three of the selected strains. Unlike the murine antibodies, the naturally elicited antibodies tolerated variation in the first five residues of FP, neutralizing strains with FP8_v2 (tolerating an Ile to Leu change at residue 515) and FP8_v3 (tolerating a Val to Ile change at residue 513) (Fig 2B).

We assessed the ability of the guinea pig sera to neutralize strains from the FP-non-matching panel. At week 28, one animal each neutralized three or four strains, two animals each neutralized two strains, whereas animal CGP701-2 did not neutralize any strains (Fig 2C, top panel). After the CH505 trimer boost, there was not a clear increase in neutralization, with two animals neutralizing three strains, and three animals neutralizing one strain. After the cocktail boost with CH505 and BG505 Env trimers, one animal could now neutralize five strains, two could neutralize four strains, another animal neutralized three strains, and a fifth animal—CGP701-2, which showed limited breadth on the FP-matching panel—neutralized none (Fig 1C). Both with FP-directed antibodies (Fig 2B) and with guinea pig immunized sera (Figs 1B and 2C), we did not observe a strong preference for neutralization of sensitive strains over resistant strains, nor of Tier 1 strains over Tier 2 strains, suggesting neutralization by FP-directed antibodies and sera to be governed more by FP8 sequence constraints than by strain-neutralization sensitivity. Overall, the additional trimer boosts increased the consistency of broad serum neutralization as assessed on the FP-nonmatching panel.

### ELISA titers plateau in response to CH505-Env trimer immunizations

**Fig 3:**
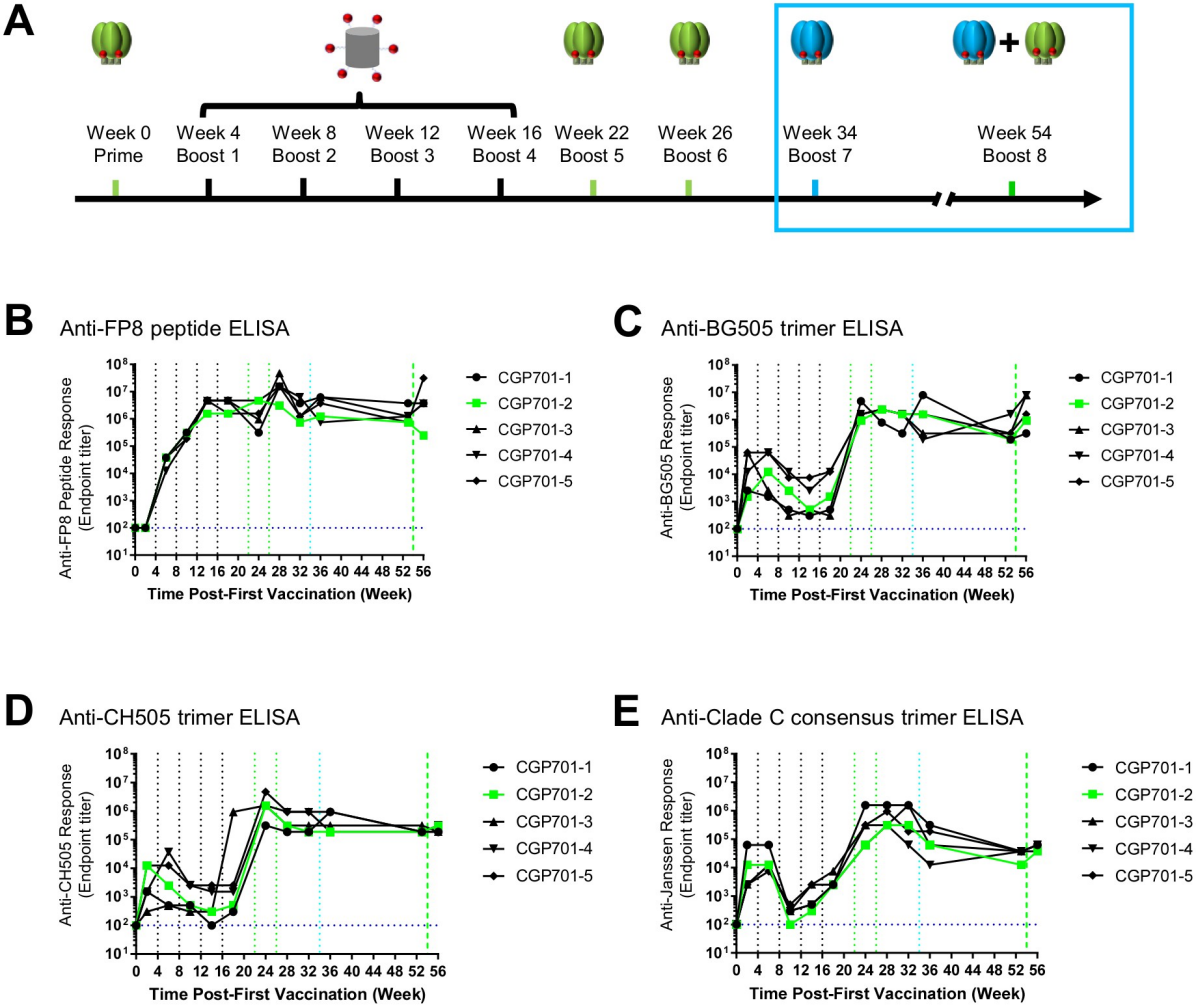
ELISA titers plateau in response to CH505 immunizations at week 34 and 54. Anti-FP and trimer responses elicited during prime and boost immunization were assessed serologically. **A**. Immunization scheme. Initial seven immunizations described in Xu et al. [13], with final two boosts (blue outline) at weeks 34 and 54 described here. Serum assessments occurred two weeks after each immunization. **B**. Anti-FP response. **C**. Anti-BG505 trimer response. **D**. Anti-CH505 trimer response. **E**. Anti-clade C consensus trimer response.

To assess how the overall immune response related to the development of neutralization breadth, we used ELISA to measure overall responses to both peptide and Env trimer (Fig 3). For the FP8 peptide, we observed ELISA titers to increase in all five animals through the fourth FP-KLH prime. A slight drop in FP ELISA titer in most animals (except with CGP701-2) was observed upon trimer boost. In general, anti-FP8 peptide responses plateaued by week 14, and did not seem to be strongly influenced by the additional CH505-Env boosts (Fig 3B).

**Fig 4:**
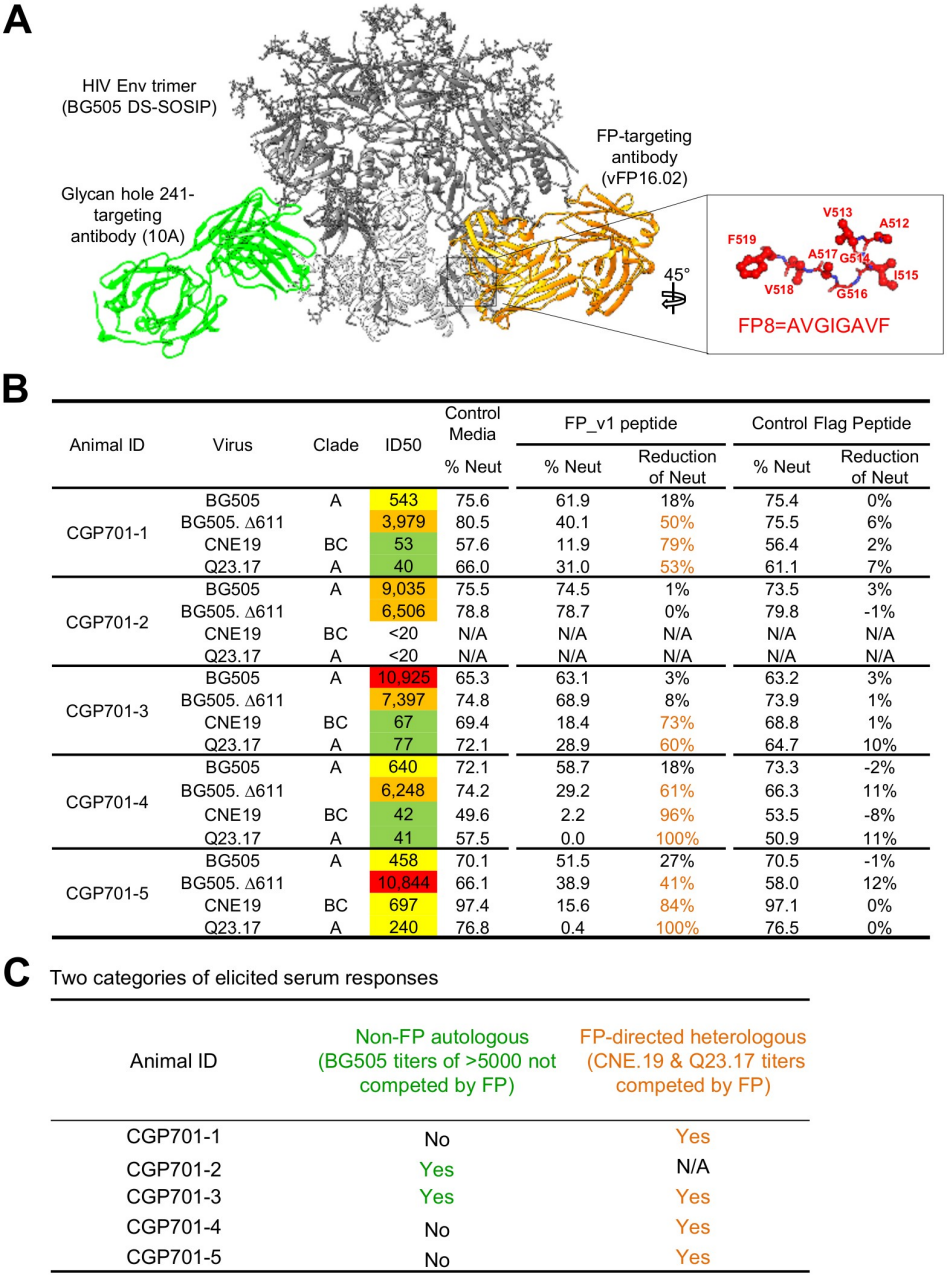
FP competition reveals two categories of immune responses: (i) autologous with high BG505 titers, not competed by FP9 peptide; and (ii) cross-clade with lower titers, competed by FP9 peptide. **A**. Examples of the two categories of elicited responses. (i) Autologous (green), directed to a BG505-specific glycan hole and (ii) broad (orange), directed to FP. **B**. FP competition in the presence of FP9 peptide or controls (Media and Flag—see Methods). The assays were repeated two to three times, with representative data of one set of repeats reported. **C**. Categories of elicited serum responses. N/A: cannot be analyzed.

For Env trimer, we assessed reactivity to BG505, to CH505, and to a consensus clade C. Overall, an early initial peak was seen in Env trimer ELISA, which then decreased in titer during additional FP-KLH immunizations, before recovering and beginning to increase by week 16 and peaking around week 24, two weeks after the BG505 trimer boost (Figs 3C and 4E). For BG505-directed ELISA, titers plateaued or slightly decreased during CH505 boosts. For CH505-directed ELISA, titers also plateaued at week 28. And for a consensus clade C trimer, titers dropped about 10-fold between week 32 and week 36, where they stayed through week 56. Thus, the increase in consistency in cross-clade neutralizing responses upon repetitive Env-trimer boosts (Figs 1 and 2) was not accompanied by an increase in ELISA titers against either FP8 peptide or various Env trimers.

### FP competition reveals two categories of immune responses

To gain insight into the regions of the HIV-Env trimer recognized by neutralizing responses, we used soluble FP9 peptide (the first 9 residues of the most prevalent fusion peptide, AVGIGAVFL) to quantify the neutralization that derived from antibodies directed against FP. We tested two BG505-neutralizing vaccine-elicited antibodies: antibody 10A, which targets a glycan hole located at residue 241 [20], and antibody vFP16.02, which targets the N terminus of the fusion peptide [13] (Fig 4A). We also tested two infection-elicited antibodies, VRC01 [21] and VRC34.01, directed at CD4-binding site and FP, respectively. Competition with free FP9 peptide showed little reduction of neutralization for antibody 10A or antibody VRC01; by contrast, competition with free FP9 peptide reduced neutralization of antibody vFP16.02 or antibody VRC34.01 by 100% (S2 Table), whereas media or control FLAG peptide had little impact on neutralization. Thus, FP competition appeared to be specific for FP-directed neutralizing responses.

We next tested the ability of free FP peptide to compete the neutralization of each of the guinea pig week 56 sera, against four viruses: (i) wild-type BG505, (ii) Δ611 BG505, (iii) strain CNE19, and (iv) strain Q23.17 (Fig 4B). We observed several types of neutralizing immune responses involving both autologous and heterologous viral strains. For wild-type BG505, the autologous strain used in immunization, serum neutralization appeared to be primarily non-FP directed; neutralization by all five sera decreased less than 30% by the addition of free FP peptide. By using the glycan Δ611-deleted version of BG505, which is more than 10-fold more sensitive to neutralization by FP-directed antibodies [13, 18], we could observe higher FP-directed neutralizing responses; these were inhibited by at least 30% in three guinea pigs (CGP701-1, -4 and -5). Neutralization of heterologous strains CNE19 and Q23.17, from the FP-matching subpanel, provided further delineation of FP-directed responses. Specifically, all of the sera except CGP701-2 neutralized these two strains, and each of these heterologous neutralizing responses was inhibited by free FP (Fig 4B).

Overall, autologous neutralization appeared to derive from a mixture of FP and non-FP-directed responses, some of which were directed at glycan holes specific to BG505 (S3 Table); heterologous neutralizing responses, however, appeared to be primarily FP-directed. Two of the animals (CGP701-2 and -3) had high autologous BG505 titers, and neither of these responses was substantially inhibited by free FP. Four of the animals (all but CGP701-2), meanwhile, showed broad heterologous neutralization—and in all four of these animals, free FP substantially inhibited neutralization. Overall, vaccine-elicited responses appeared to fall into two categories: (i) high-titer autologous, but not FP-directed and (ii) moderate-titer heterologous and FP-directed (Fig 4C), and immunized guinea pigs in our study had either or both types of response.

### Autologous and heterologous responses develop at different rates and to different overall levels

**Fig 5:**
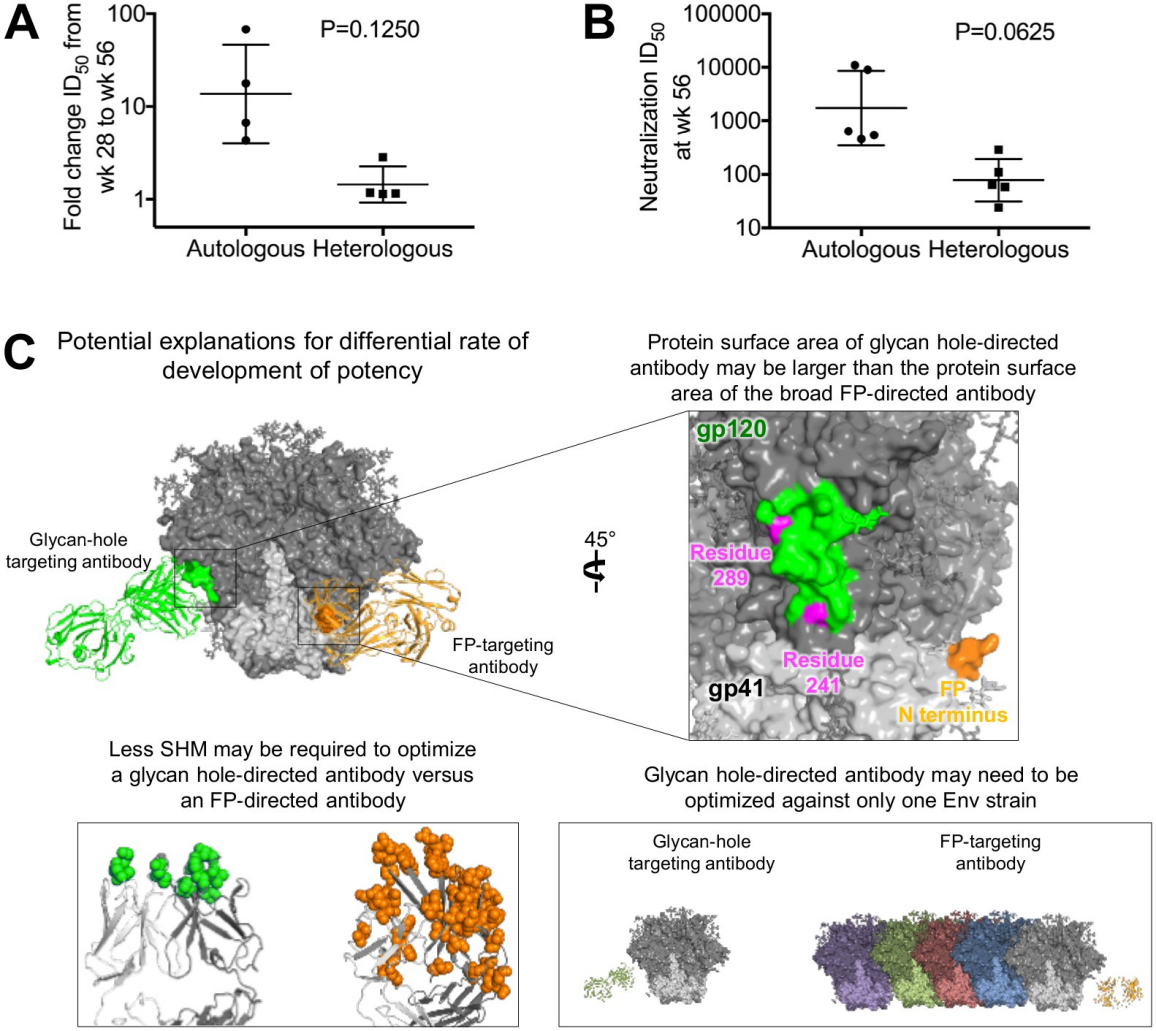
Autologous neutralizing responses can develop to high potency after a single boost, whereas the broad FP-directed response generally develop more slowly, requiring multiple boosts. **A**. Autologous neutralizing response increased to high titer while heterologous neutralizing response (calculated from the geometric mean of the 18 heterologous strains in Figs 1B and 2C) developed more slowly from week 28 to week 56. Strains with a value of “<20” were excluded from geometric mean calculation. **B**. Autologous neutralizing responses were more potent than heterologous responses. **C**. Potential mechanistic explanations for the more rapid increase and higher overall titer of autologous neutralizing responses [atomic-level analyses were not possible for glycan-hole directed antibodies, as structural definition of their interactions have only been determined at low resolution (e.g. [20])].

We further analyzed the developmental rate and overall magnitude of the vaccine-induced neutralizing responses. These tended to be higher for autologous responses, and lower for heterologous responses (Fig 5). Potent autologous neutralizing responses developed between week 36 and week 56, for example with guinea pigs CGP701-2 and CGP701-3, for which BG505 titers were under 1000 ID_50_ at week 36 and, in responses to a single cocktail boost, rose to ~10,000 ID_50_ by week 56, with an increase in titer of over 10-fold (Fig 1B). By contrast, the highest heterologous FP-directed response (matching FP8-sequence) at week 56 was just over 1,000 for CGP701-5, when assessed against the clade C strain 25710. While fold-change in titer between week 28 and week 56 trended to significance (P = 0.1250) (Fig 5A), this trend could be explained in part by the higher overall potency for autologous versus heterologous responses at week 56 (Fig 5B). Potential explanations involve differences in developmental rate for autologous glycan-hole-targeting antibodies versus heterologous FP-directed antibodies, such as differences in recognized protein surface between glycan-hole-targeting and FP-targeting antibodies, lower somatic hypermutation (SHM) requirements for glycan-hole neutralization versus FP, or difficulties in targeting broad responses against multiple viruses versus against a single virus (Fig 5C).

### Guinea pig serum neutralization correlates with neutralization by known FP-directed antibodies

**Fig 6:**
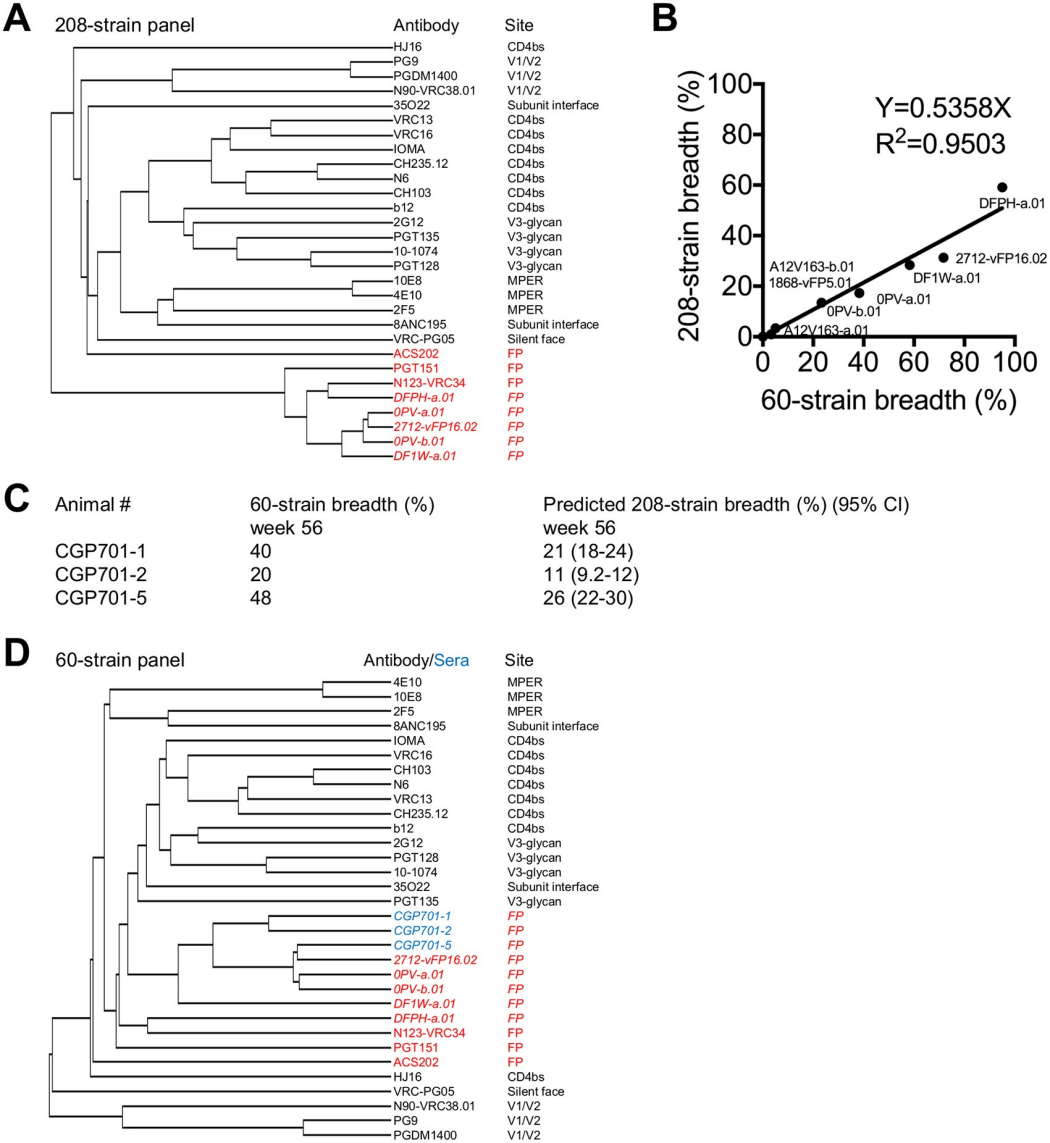
Estimated breath of guinea pig sera. **A**. FP-directed neutralizing antibodies cluster together with similar neutralization fingerprints [22] as assessed on 208 strains. Vaccine-elicited antibodies are shown in italics with FP-directed antibodies highlighted in red. **B**. Correlation of breadths for FP-directed antibodies on 60-strain panel versus 208-strain panel. 95% CI of slope ranges from 0.4604 to 0.6112. **C**. Guinea pig serum neutralization breadth on 60-strain panel and predicted breadth on 208-strain panel based on correlation in B, with ID_50_ shown in S4 Table. Predicted breadths are shown with 95% CI in parentheses. Sufficient sera were available to allow neutralization characterization with only the three animals shown. **D**. Neutralization fingerprints cluster together [22] for guinea pig sera (CGP701-1, CGP701-2, CGP701-5, highlighted in blue) and FP-directed neutralizing antibodies (highlighted in red) when assessed on a 60-strain panel. Notably, the vaccine-elicited antibodies and sera (shown in italics) cluster as a separate subgroup.

As epitope specificities of HIV-1–neutralizing antibodies in serum can be elucidated from the serum pattern of neutralization against a diverse panel of HIV-1 isolates [22], we sought to compare the neutralization of guinea pig serum with neutralization by known antibodies. We thus analyzed neutralization fingerprints of known antibodies, using one representative class member from each of the different classes of broadly neutralizing antibodies currently known to recognize Env trimer [23]. We added to this basis set of broadly neutralizing antibodies representative members from the vaccine-elicited murine vFP1.01 class (2712-vFP16.02) as well as from the vaccine-elicited classes induced by vaccination of rhesus macaques, DF1W, 0PV and DFPH [24]. This neutralization fingerprint analysis showed most of the FP-directed antibodies to form a tight cluster (Fig 6A).

To compare the similarity between guinea pig sera and known antibodies, optimally we would assess neutralization on our 208-strain panel, as this provides detailed information on breadth and potency over a diverse range of HIV-1 strains. However, we were limited by the volume of serum available to test neutralization. We had sufficient sera from three animals at week 56 to assess their neutralization on a 60-strain panel (S4 Table). In additional to the neutralization on 60 strains, we also tested the reproducibility of the neutralization by FP competition on two Tier 2/R viruses, 263–8 and ZM106.9 (S6 Table); we observed that the presence of FP reduced neutralization by greater than 30% for the sera, including CGP701-2 whose serum neutralization against 263–8 was reduced by 46%.

Overall, correlation between the 60-strain and 208-strain panels was strong (R^2^ = 0.95, Fig 6B), substantially higher than the correlation between the 19-strain and 208-strain panels (R^2^ = 0.64). Based on the 60-strain panel correlation, animals CGP701-1, CGP701-2 and CGP701-5 had estimated 208-strain breadths of 21%, 11% and 26%, respectively (Fig 6C).

We further assessed the pattern of neutralization between known antibodies and the guinea pig sera by using neighbor-joining analysis to create a neutralization fingerprint dendrogram that mixed neutralization by monoclonal antibodies and by sera (Fig 6D). All three guinea pig sera clustered with known FP-directed antibodies, and most closely with those that were vaccine elicited. Unexpectedly, CGP701-1 and CGP701-2 clustered closely, despite the absence of heterologous neutralization for CGP701-2 on the 19-strain panel. Inspection of neutralization on the 60-strain panel (S4 Table) revealed clear similarities in strains neutralized by the three guinea pig sera. This is consistent with the observation that CGP701-2 serum neutralization on virus 263–8 was competed by free FP (S6 Table). These results indicate that all five guinea pigs to have a characteristic FP-directed neutralizing response.

With CGP701-2, cross-clade neutralization was not apparent on the 19-strain panel, and the characteristic FP-directed response became apparent only with the larger 60-strain panel. With CGP701-4, cross-clade neutralization could be seen with seven heterologous strains neutralized on the 19-isolate panel. Finally, with three of the animals, CGP701-1, -3, and -5, broad cross-clade neutralization was apparent in the 19-strain panel, with neutralization breadths for two of these estimated from the 60-strain panel to be 20–30% on the 208-strain panel. While estimating 208-strain panel neutralization from small panel data is not as reliable as directly measuring 208-strain neutralization, the high correlation we observed between 60-strain and 208-strain panels did allow for meaningful extrapolation.

## Discussion

The vaccine elicitation of sera capable of neutralizing diverse strains of HIV-1 has been a long-sought goal. Tantalizing neutralization results have been achieved against the FP site of HIV-1 vulnerability. However, these results needed improvements in breadth, potency, and consistency. Here we show that additional Env trimer boosting can enhance the consistency of neutralization titers in animals previously primed with FP coupled to a carrier protein. Prior to additional boosting, broad responses were clearly seen in only one animal (CGP701-5) on a 19-strain panel, and after boosting in three of five animals: CGP701-1, -3 and -5. Furthermore, after assessment on a 60-strain panel, fingerprint analysis could detect evidence of a characteristic FP-directed response, even in the animal CGP701-2, which showed no evidence of heterologous response on the 19-strain panel.

We note that the neutralization titers for some animals were relatively low. However, the neutralization assay we used has been well characterized and shown to provide reproducible measurements of neutralization above 20 ID_50_ [25]. In the 15 measured sera (three time points for five animals) we did observe clear non-specific neutralization with three sera: CGP701-1 at week 28, CGP701-5 at weeks 28 and 56. For CGP701-1, we observed the neutralization on the 19-strain panel to be higher at weeks 36 and 56, where we did not observe background neutralization, indicating that the background at week 28 did not substantially alter the measured HIV-1 neutralization. For CGP701-5, we set a threshold of 50 ID_50_ for positive assessment of neutralization, as this was higher than the non-specific neutralization observed with SIVmac251.

The increase in titer against heterologous strains with the FP-matching subpanel occurred gradually—being detectable (>20 ID_50_) in 12 out of 45 tested neutralizations at week 28, in 20 out of 45 at week 36, and in 31 out of 45 at week 56 (Fig 1C). For the FP-nonmatching subpanel, increases were less general—being detectable in 12 out of 45 tested neutralizations at week 28, in 13 out of 45 at week 36, and in 18 out of 45 at week 56 (Fig 2C). A large jump in potency between week 36 and 56 was observed with one of the strains of the FP-nonmatching panel, strain TH023. We also note that none of the two strains with FP8_v3 had detectable neutralization (Fig 2C), although a meaningful correlation between neutralized strains and FP sequences would require a larger number of viruses of each FP sequence to be assessed. Overall, it remains to be determined the parameters that govern the rate of increase in potency.

Because strain BG505 is missing a glycan at position 241, close to the FP site of vulnerability, we additionally assessed whether the increase in breadth could be linked specifically to neutralization of strains with glycan *N*241, as we had boosted with strain CH505, which has a full set of glycans around FP including glycan *N*241. The 19-tested strains include 15 strains with *N*241 and 4 strains lacking *N*241; overall, neutralization breadth generally increased with CH505 boost, though without a clear dependence on the presence (or absence) of glycan 241 (S2 Fig). We note that CH505 is one of the strains of trimer with complete glycan shield, an attribute shown to relate to elicitation of antibodies with high neutralization breadth [26].

Overall, our results with additional Env trimer boosting of animals previously immunized with FP-carrier/BG505 Env trimer provide the first consistent elicitation in guinea pigs of broad HIV-1 neutralizing responses. The CH505 Env trimer appeared to boost previously induced responses, raising these above the detection threshold (<20 ID_50_). Notably, the increase in breadth induced by the CH505 Env trimer boost occurred even though the chimeric CH505 Env used in this study had the same FP8 sequence as both FP8-KLH and BG505; thus, the CH505-induced increase in breadth likely occurred through increased recognition of regions of Env trimer in general, not through increased recognition of the N-terminus of FP in specific. The lack of correlation between ELISA responses to FP, BG505 trimer, CH505 trimer or a consensus clade C trimer and the elicited neutralization breadth suggests a critical parameter may be appropriate maturation of the response, but most likely not the overall titer of reactive antibody. It remains to be seen whether additional Env trimer boosting—or boosting with other immunogens such as Env nanoparticles or Env VLPs—can further increase neutralization titer and breadth.

## Materials and methods

### Animal protocols and immunization

Animals were housed and cared for in accordance with local, state, federal, and institute policies in an American Association for Accreditation of Laboratory Animal Care-accredited facility at Vaccine Research Center (VRC), NIAID, NIH. Approval of this study by the VRC Animal Care and Use Committee ensured compliance with Animal Welfare Act requirements for environment enhancement adequate to promote the psychological and physical well-being of small animals. Specific humane interventions are described in the animal care protocol, including detailed descriptions of monitoring frequency, use of anesthesia and analgesia for procedures, and humane method of euthanasia based upon the American Veterinary Medical Association (AVMA) Guidelines on Euthanasia, or as recommended by the veterinary staff. Female Hartley guinea pigs with body weights of 300g were obtained from Charles River Laboratories (Wilmington, MA) and used for immunization studies. They were co-housed in pairs in Allentown NexGen 1800 micro-isolator cages with 280 in^2^ floor space. All animals were provided ad-lib feed Guinea Pig Diet (Lab Diet, St. Louis, MO) with supplemental food items such as oranges and reverse osmosis water following the “Guide for the Care and Use of Laboratory Animals, 8th Edition” (National Research Council, 2011). Environment enrichment includes social housing, bedding/nesting material and dumbbell toys. Animals were monitored twice daily (once daily on weekends/holidays) and received physical exams weekly during cage change procedures. During the physicals the animals were thoroughly palpated checking for any abnormalities, the animals’ extremities, ears and snout were checked for good pigmentation, and nails were trimmed as required. If any abnormalities were found, the veterinarian was consulted and medication provided as determined by the veterinarian. For each immunization, 0.4 ml immunogen mix, containing 25 μg of filter-sterilized immunogen and 80 μl of Adjuplex (Sigma-Aldrich Inc, MO or Adjuplex equivalent formulated based on US Patent 6,676,958 B2) in PBS, were injected via a needle syringe to the caudle thigh of the two hind legs.

### Cell lines

Expi293F cells were from ThermoFisher Scientific Inc (Invitrogen, cat# A14528; RRID: CVCL_D615). HEK 293T/17 cells were from ATCC (cat# CRL-11268^™^). TZM-bl cells were from NIH AIDS Reagent Program (www.aidsreagent.org, cat# 8129).

### FP-KLH immunogens

HIV-1 fusion peptide (FP8: AVGIGAVFC, FP7: AVGIGAVC, FP6: AVGIGAC, FP5: AVGIGAC) were synthesized (GenScript, Piscataway, NJ) with a free amine at the N terminus and an extra cysteine residue appended to the C terminus. KLH conjugates were prepared via in two steps. The first is activation of carrier protein keyhole limpet hemocyanin (KLH; Thermo-Scientific) using m-maleimidobenzoyl-N-hydroxysuccinimide ester (MBS), this was followed by coupling of terminal thiol to the maleimide of the activated KLH. The antigenicity of the conjugates was confirmed by binding of fusion peptide specific antibodies VRC34.01, PGT151 and ACS202.

### HIV-1 envelope trimer

Non-tagged HIV Env trimers (BG505.DS-SOSIP, CH505-chim.DS-SOSIP, Clade C consensus trimer) were produced in transiently transfected 293F cells as previously described [27–29]. Briefly, the plasmid encoding the trimer and the plasmid encoding human furin were mixed at 4:1 ratio and used to transfected 293F cells at 0.75 mg plasmid / 1 L cells (1.5 x 10^6^ /ml) using 293Fectin (Thermo Scientific) or Turbo293 transfection reagent (Speed BioSystems). Cells were incubated in shaker at 120 rpm, 37 °C, 9% CO_2_. On the next day of transfection, 80 ml HyClone SFM4HEK293 medium and 20 ml FreeStyle 293 Expression Medium were added to each liter of cells. The native-like Env trimer protein was purified from the day-7 supernatant with 2G12 or VRC01 affinity chromatography, followed by gel filtration on a Sephadex200 16/60HL column and negative selection with a 447-52D affinity column to remove V3-exposed trimers. The antigenicity of the trimers was confirmed with a panel of antibodies in the Meso Scale Discovery (MSD) platform. For sorting probes, the avi-tagged trimers were further biotinylated using the BIRA500 kit (Avidity, LLC) and purified by gel filtration chromatography.

### Anti-trimer (BG505 DS-SOSIP.664 or CH505-chim DS-SOSIP.664 or clade C consensus trimer) Enzyme-Linked Immunosorbent Assay (ELISA)

Anti-trimer ELISA was modified based on previously reported method with lectin captured trimer [30]. Ninety-six-well plates (Costar High Binding Half-Area; Corning, Kennebunk, ME) were coated overnight at 4°C with 50 μl/well of 2 μg/ml snowdrop lectin from *Galanthus nivalis* (Sigma-Aldrich, St. Louis, MO) in PBS. Between each subsequent steps, plates were washed five times with PBS-T (PBS plus 0.05% Tween). After being coated, plates were blocked with 100 μl/well of blocking buffer (5% skim milk in PBS) and incubated at room temperature for 60 min, followed by trimer capture with 50 μl/well of 2 μg/ml trimer proteins in 10% FBS-PBS for 2 hours at room temperature. Next, 50 μl/well serially diluted (5-fold; starting dilution, 1:100) monkey plasma in 0.2% Tween-PBS buffer was added and incubated for 1 hour at room temperature. Afterward, goat anti-guinea pig IgG antibody conjugated to horseradish peroxidase (KPL, Gaithersburg, MD) diluted 1:5,000 in 0.2% Tween-PBS buffer was added at 50 μl/well for 60 min. Plates were washed five times with PBS-T and developed with 50 μl/well tetramethylbenzidine (TMB) substrate (SureBlue; KPL, Gaithersburg, MD) for 10 min before the addition of 50 μl/well 1 N sulfuric acid (Fisher Chemical, Fair Lawn, NJ), without washing, to stop the reaction. Plates were read at 450 nm (SpectraMax using SoftMax Pro, version 5, software; Molecular Devices, Sunnyvale, CA), and the optical densities (OD) were analyzed following subtraction of the nonspecific horseradish peroxidase background activity. The endpoint titer was defined as the reciprocal of the greatest dilution with an OD value above 0.1 (2 times average raw plate background).

### Anti-fusion peptide ELISA

Animal sera and monoclonal antibodies were assessed for binding to a biotinylated eight amino acid linear peptide FP8-PEG12-biotin (GenScript, Piscataway, NJ). Streptavidin coated plates (Thermoscientific, Rockford, IL) were washed with PBS-T buffer (10% Tween-20 in 1X PBS) then incubated with FP8v1-PEG12-biotin at 37 °C for 2 hours. Animal sera were heat-inactivated at 56 °C for 1 hour and assessed at 7-point 4-fold dilutions starting at 1:25 dilutions. Monoclonal antibodies were tested at 7-point 5-fold dilutions starting at 5 μg/ml. Samples were first incubated on plates at 37 °C for 1 hour, then the plates were washed with PBS-T buffer. Secondary antibody (goat anti-guinea pig IgG HRP) was added and incubated in wells at 37 °C for 1 hour before the final PBS-T buffer wash. TMB substrate (SureBlue, KPL, Gaithersburg, MD) was equilibrated to ambient temperature and incubated in wells for 10 minutes, when the reaction was stopped with 1 N sulfuric acid. The plates were then read on a microplate spectrophotometer (Biotek Epoch, Winooski, VT) and the endpoint titer for each sample was determined.

### Neutralization assays

A single round virus infection assay using TZM-bl target cells was performed to assess antibody neutralization as described [18]. Briefly, 293T-derived HIV-1 Env-pseudotyped virus stocks were generated by cotransfection of an Env expression plasmid and a pSG3ΔEnv backbone. Animal sera were heat-inactivated at 56 °C for 1 hour and assessed at 8-point 4-fold dilutions starting at a 1:20 dilution. Monoclonal antibodies were tested at 8-point 5-fold dilutions starting at 50 μg/ml or 500 μg/ml. Virus stocks and antibodies (or sera) were mixed in a total volume of 50 μl and incubated at 37 °C for 1 hr. TZM-bl cells (20 μl, 0.5 million/ml) were then added to the mixture and incubated at 37 °C. Cells were fed with 130 μl cDMEM on day 2, lysed and assessed for luciferase activity (RLU) on day 3. Percentage neutralization was calculated in comparison to virus only controls with cDMEM in place of serum or antibody. A nonlinear regression curve was fit using 5-parameter hill slope equation. The 50% and 80% inhibitory dilutions (ID50 and ID80) were determined for sera and the 50% and 80% inhibitory concentrations (IC50 and IC80) were determined for monoclonal antibodies.

Neutralization with peptide competition was performed as described [13, 18]. Briefly, animal sera and monoclonal antibodies were tested at a single-point dilution that resulted in >50% neutralization of the corresponding viruses. 10 μl of animal sera or monoclonal antibodies were first mixed with 5 μl of control media, PEGylated FP9 (AVGIGAVFL) or PEGylated non-cognate FLAG peptide. The peptide/antibody mixture was incubated at 37 °C for 30 minutes before 35 μl of viruses was added. The final concentration of the peptide in the 50 μl mixture is 12.5 pM. The virus/antibody/peptide mixture was incubated at 37 °C for 30 minutes. After that, TZM-bl cells were added, incubated, fed and lysed as described above in the standard neutralization assay. The peptide competition assays were repeated two to three times with representative results of one set of repeats reported.

### Statistical analyses

Linear regression with line through the origin was performed for Fig 6B. Wilcoxon matched-pairs signed ranked test was performed in Fig 5A and 5B. All statistical tests were performed using the statistical package R.

### Neutralization fingerprinting analysis

The neutralization fingerprint of a monoclonal antibody is defined as the potency pattern with which the antibody neutralizes a set of diverse viral strains. The neutralization fingerprints of FP-targeting antibodies identified in this paper and a set of published monoclonal antibodies were compared and clustered according to fingerprint similarity, as described previously [22]. Neutralization data of 208 HIV-1 viral strains were used in the fingerprint analysis.

### Ethics statement

All animal experiments were reviewed and approved in protocol VRC-16-552 by the Animal Care and Use Committee of the Vaccine Research Center, National Institutes of Allergy and Infectious Diseases (NIAID), National Institutes of Health (NIH) and all animals were housed and cared for in accordance with local state, federal and institute policies in an American Association for Accreditation of Laboratory Animal Care-accredited facility with stringent standard operating procedures and compliant with *U*.*S*. *Animal Welfare Act (AWA) and Regulations*, the *Public Health Service (PHS) Policy on Humane Care and Use of Laboratory Animals*, the *Guide for the Care and Use of Laboratory Animals* and all applicable NIH Policies on in vivo research. Animal procedures were conducted in strict accordance with all relevant federal and National Institutes of Health guidelines and regulations.

## Supporting information

S1 Table HIV-1 strain tier designation versus antibody neutralization sensitivity. As different HIV-1 strains differ in their susceptibility to antibody neutralization, when assessing neutralizing responses, it is critical to know the general neutralization susceptibility of tested viruses. “Tier 1” viruses comprise “easy” to neutralize open viruses often obtained after serial laboratory passage, and “Tier 2” viruses comprise “difficult’ to neutralize closed viruses typical of the strains transmitted by natural infection. Since some of the tested viruses in our 208-strain panel [23] have not been assigned a tier, we assessed their neutralization sensitivity to antibodies known to neutralize preferentially Tier 1 strains. We tested two classifications: one involving antibodies 17b, 48d and F105 (A), and a second involving these antibodies along with antibodies directed at V3, antibodies 447-52D and 3074 (B). In (A), we found high discrimination for resistant strains, but not for sensitive strains, whereas in (B), the discrimination was more balanced. We thus decided to use the classification shown in (B).
(PDF)

S2 Table FP competition of virus neutralization by FP-directed and non-FP-directed antibodies. FP peptide did not compete with CD4-binding site antibody VRC01 or 241, 289 glycan hole-targeted rabbit antibody 10A, but competed with FP-directed antibody VRC34.01 or vFP16.02. Antibody neutralizing activity against BG505 Δ611 virus was measured in the absence (Media only) or in the presence of 25 μg/ml of FP or control FLAG peptide. Reduction of neutralization (Neut) was calculated relative to Media.
(PDF)

S3 Table Mapping of autologous BG505 neutralization to glycan holes at 241 or 289. Neutralizing activity was measured as ID_50_ for each indicated guinea pig serum against BG505 virus, or BG505 S241N or P291S mutants, which restored *N*241 or *N*289 glycan, colored similarly as in Figs 1 and 2. A reduction of serum-neutralizing activity on glycan-restored mutants relative to glycan-intact BG505 virus that was larger than 50% was defined as significant (highlighted in green).
(PDF)

S4 Table Serum neutralization on a 60-strain panel of diverse HIV-1 viruses. Week 56 guinea pig sera at serial dilutions were assessed for virus neutralization, and the ID_50_ values were calculated. SIVmac251 was used as a negative control (light-gray shaded). ID_50_ values ≥30 were considered neutralizing, except for CGP701-5, where only those with ID_50_ ≥50 were considered positive. Entries are colored according to the color keys on the right. Tier status defined by sensitivity to *HIV-IG* [19]. HIV-1 strains were further categorized as resistant (R) or sensitive (S) based on their neutralization by five antibodies: 17b, 48b, F105, 3074 and 447-52D, which generally only neutralize open Tier 1 strains. However, as described in the legend to S1 Table, antibody 3074 does neutralize some Tier 2 strains with an IC_50_ of less than 50 μg/ml, and thus an IC_80_ of above 50 was used—and these have been marked with an asterisk.
(PDF)

S5 Table Non-HIV virus neutralization assessments. All samples were assessed for neutralization against non-HIV viruses, SIVmac251 and SVA-MLV, and ID_50_ values are listed. As can be seen, sporadic neutralization of non-HIV-1 viruses was observed. Based on these results, we considered neutralization titers positive for HIV-1 when ID_50_ ≥30 for CGP701-1 through CGP701-4 or when ID_50_ ≥50 for CGP701-5. We note that despite some non-specific neutralization by CGP701-5 sera, the neutralization fingerprint of this sera clustered most closely with the vaccine-elicited FP-directed antibodies (Fig 6), providing strong evidence that the observed neutralization by CGP701-5 was FP directed and not a background artifact.
(PDF)

S6 Table Fusion peptide competition of neutralization of two Tier 2 viruses. Sufficient sera from animals CGP701-1, -2, and -5 were available to assess FP competition for two Tier 2/R viruses from the 60-strain panel (S4 Table). Percentage reduction of neutralization was calculated by comparing the percentage neutralization in the presence of soluble FP9 peptide with that in the presence of media. Control assays were performed side-by-side using a Flag peptide. The assays were repeated two to three times, with representative data of one set of repeats reported. The assay results confirmed the reliability of the neutralization assays. Soluble FP could compete substantially, >30%, the neutralization by all three sera, except for virus 263–8 by CGP701-5 serum with 18% reduction in the presence of FP. The CGP701-5 serum had an ID_50_ of 44 against 263–8, below the cutoff titer of 50 for this serum (S5 Table).
(PDF)

S1 Fig Representative neutralization curves of guinea pig sera against HIV-1 and control viruses. Guinea pig sera at week 56 post immunization were diluted at indicated reciprocal dilution starting from 1:20. Percentage neutralization was calculated by comparing with blank controls without serum. ID50 values were determined at 50% neutralization (dashed lines) by nonlinear regression curve fitting as detailed in the Method section.
(PDF)

S2 Fig Development of neutralization breadth upon CH505 trimer boost for glycan 241-containing and glycan 241-missing viruses. **A**. Serum neutralization breadth of glycan 241-containing and glycan 241-missing viruses for animals CGP701-1 to CGP701-5 at different time points. **B**. Differences in neutralization breadth for glycan 241-containing and glycan 241-missing viruses from different time points. Strains containing glycan 241: T278, 6644, 6405, A03349M1, ADA, C4118, TH023, BI369, KER2008, Q23, 3988, BL01, 286, 0815, and CNE19. Strains not containing glycan 241: BG505, CNE56, 25710, and 0077.
(PDF)
