# Supplementary material for: Consistent elicitation of cross-clade HIV-neutralizing responses achieved in guinea pigs after fusion peptide priming by repetitive envelope trimer boosting
Source: PLoS One. 2019 Apr 17;14(4):e0215163. doi: 10.1371/journal.pone.0215163 (PMC6469787; doi:10.1371/journal.pone.0215163)
Supplement: S1 Table — As different HIV-1 strains differ in their susceptibility to antibody neutralization, when assessing neutralizing responses, it is critical to know the general neutralization susceptibility of tested viruses. “Tier 1” viruses comprise “easy” to neutralize open viruses often obtained after serial laboratory passage, and “Tier 2” viruses comprise “difficult’ to neutralize closed viruses typical of the strains transmitted by natural infection. Since some of the tested viruses in our 208-strain panel [23] have not been assigned a tier, we assessed their neutralization sensitivity to antibodies known to neutralize preferentially Tier 1 strains. We tested two classifications: one involving antibodies 17b, 48d and F105 (A), and a second involving these antibodies along with antibodies directed at V3, antibodies 447-52D and 3074 (B). In (A), we found high discrimination for resistant strains, but not for sensitive strains, whereas in (B), the discrimination was more balanced. We thus decided to use the classification shown in (B). (PDF) [file pone.0215163.s001.pdf]

S1 Table. HIV-1 strain tier designation versus antibody neutralization sensitivity. As different HIV-1 strains differ in their susceptibility to antibody neutralization, when assessing neutralizing responses, it is critical to know the general neutralization susceptibility of tested viruses. “Tier 1” viruses comprise “easy” to neutralize open Env viruses often obtained after serial laboratory passage, and “Tier 2” viruses comprise “difficult’ to neutralize closed Env viruses typical of the strains transmitted by natural infection. Since some of the tested viruses in our 208-strain panel [23] have not been assigned a tier, we assessed their neutralization sensitivity to antibodies known to neutralize preferentially Tier 1 strains. We tested two classifications: one involving antibodies 17b, 48d and F105 (A), and a second involving these antibodies along with antibodies directed at V3, antibodies 447-52D and 3074 (B). In (A), we found high discrimination for resistant strains, but not for sensitive strains, whereas in (B), the discrimination was more balanced. We thus decided to use the classification shown in (B).

A. Number of sensitive or resistant strains out of the 152 in the 208-strain panel that have a tier designation, based on resistance criterion: IC_{50} >50 μg/ml for antibodies 17b, 48d, and F105. Fisher’s exact test: $P<0.0001$.

Tier designation	Sensitive	Resistant
2, 2/3, 3, 1/2, 1B/2	4	136
1,1A,1B	7	5

B. Number of sensitive or resistant strains out of the 152 in the 208-strain panel that have a tier designation, based on resistance criterion: IC_{50} >50 μg/ml for antibodies 17b, 48d, F105, and 447-52D; and IC_{80} >50 μg/ml for antibody 3074. Fisher’s exact test: $P<0.0001$.

Tier designation	Sensitive	Resistant
2, 2/3, 3, 1/2, 1B/2	20	120
1,1A,1B	9	3
