# Supplementary material for: Consistent elicitation of cross-clade HIV-neutralizing responses achieved in guinea pigs after fusion peptide priming by repetitive envelope trimer boosting
Source: PLoS One. 2019 Apr 17;14(4):e0215163. doi: 10.1371/journal.pone.0215163 (PMC6469787; doi:10.1371/journal.pone.0215163)
Supplement: S2 Table — FP peptide did not compete with CD4-binding site antibody VRC01 or 241, 289 glycan hole-targeted rabbit antibody 10A, but competed with FP-directed antibody VRC34.01 or vFP16.02. Antibody neutralizing activity against BG505 Δ611 virus was measured in the absence (Media only) or in the presence of 25 μg/ml of FP or control FLAG peptide. Reduction of neutralization (Neut) was calculated relative to Media. (PDF) [file pone.0215163.s002.pdf]

**S2 Table. FP competition of virus neutralization by FP-directed and non-FP-directed antibodies.** FP peptide did not compete with CD4-binding site antibody VRC01 or 241, 289 glycan hole-targeted rabbit antibody 10A, but competed with FP-directed antibodies VRC34.01 and vFP16.02. Antibody neutralizing activity against BG505 Δ611 virus was measured in the absence (Media only) or in the presence of 25 μg/ml of FP or control FLAG peptide. Reduction of neutralization (Neut) was calculated relative to Media.

| Antibodies | Virus | Media | Addition of FP | | Addition of FLAG peptide | |
|---|---|---|---|---|---|---|
| | | % Neut | % Neut | Reduction of Neut | % Neut | Reduction of Neut |
| VRC01 | BG505.N611Q | 69 | 71 | -3% | 67 | 3% |
| 10A | BG505.N611Q | 66 | 71 | -7% | 63 | 4% |
| VRC34.01 | BG505.N611Q | 61 | 0 | 100% | 62 | 0% |
| vFP16.02 | BG505.N611Q | 88 | 0 | 100% | 75 | 16% |
