# Supplementary material for: Consistent elicitation of cross-clade HIV-neutralizing responses achieved in guinea pigs after fusion peptide priming by repetitive envelope trimer boosting
Source: PLoS One. 2019 Apr 17;14(4):e0215163. doi: 10.1371/journal.pone.0215163 (PMC6469787; doi:10.1371/journal.pone.0215163)
Supplement: S3 Table — Neutralizing activity was measured as ID50 for each indicated guinea pig serum against BG505 virus, or BG505 S241N or P291S mutants, which restored N241 or N289 glycan, colored similarly as in Figs 1 and 2. A reduction of serum-neutralizing activity on glycan-restored mutants relative to glycan-intact BG505 virus that was larger than 50% was defined as significant (highlighted in green). (PDF) [file pone.0215163.s003.pdf]

S3 Table. Mapping of autologous BG505 neutralization to glycan holes at 241 or 289. Neutralizing activity was measured as ID_{50} for each indicated guinea pig serum against BG505 virus, or BG505 S241N or P291S mutants, which restored glycans at *N*241 or *N*289, colored similarly as in Figs. 1 and 2. A reduction of serum-neutralizing activity on glycan-restored mutants relative to glycan-hole BG505 virus that was larger than 50% was defined as significant (highlighted in green).

Virus	BG505	BG505 S241N	Reduction	*N*241 glycan hole targeted	BG505 T332N	BG505 T332N P291S	Reduction	*N*289 glycan hole targeted	*N*241 or *N*289 glycan hole targeted
CGP701-1	649	118	82%	Yes	149	72	52%	Yes	Yes
CGP701-2	6,932	5,592	19%	No	5,183	2,497	52%	Yes	Yes
CGP701-3	8,189	8,801	-7%	No	5,875	4,094	30%	No	No
CGP701-4	598	219	63%	Yes	270	260	4%	No	Yes
CGP701-5	294	112	62%	Yes	370	90	76%	Yes	Yes
