# Supplementary material for: Consistent elicitation of cross-clade HIV-neutralizing responses achieved in guinea pigs after fusion peptide priming by repetitive envelope trimer boosting
Source: PLoS One. 2019 Apr 17;14(4):e0215163. doi: 10.1371/journal.pone.0215163 (PMC6469787; doi:10.1371/journal.pone.0215163)
Supplement: S4 Table — Week 56 guinea pig sera at serial dilutions were assessed for virus neutralization, and the ID50 values were calculated. SIVmac251 was used as a negative control (light-gray shaded). ID50 values ≥30 were considered neutralizing, except for CGP701-5, where only those with ID50 ≥50 were considered positive. Entries are colored according to the color keys on the right. Tier status defined by sensitivity to HIV-IG [19]. HIV-1 strains were further categorized as resistant (R) or sensitive (S) based on their neutralization by five antibodies: 17b, 48b, F105, 3074 and 447-52D, which generally only neutralize open Tier 1 strains. However, as described in the legend to S1 Table, antibody 3074 does neutralize some Tier 2 strains with an IC50 of less than 50 μg/ml, and thus an IC80 of above 50 was used—and these have been marked with an asterisk. (PDF) [file pone.0215163.s004.pdf]

S4 Table. Serum neutralization on a 60-strain panel of diverse HIV-1 viruses. Week 56 guinea pig sera at serial dilutions were assessed for virus neutralization, and the ID_{50} values were calculated. SIVmac251 was used as a negative control (light-gray shaded). ID_{50} values ≥30 were considered neutralizing, except for CGP701-5, where only those with ID_{50} ≥50 were considered positive. Entries are colored according to the color keys on the right. Tier status defined by sensitivity to *HIV-IG* [19]. HIV-1 strains were further categorized as resistant (R) or sensitive (S) based on their neutralization by five antibodies: 17b, 48b, F105, 3074 and 447-52D, which generally only neutralize open Tier 1 strains. However, as described in the legend to S1 Table, antibody 3074 does neutralize some Tier 2 strains with an IC_{50} of less than 50 µg/ml, and thus an IC_{80} of above 50 was used – and these have been marked with an asterisk.

				ID50			IC50 (µg/ml)				
**Virus ID**	**Clade**	**Tier**	**Sensitivity**	**CGP701-1**	**CGP701-2**	**CGP701-5**	**17b**	**48D**	**F105**	**447-52D**	**3074**
246-F3.C10.2	AC	2	R	37	27	84	>50	>50	>50	>50	>50
Q259.17	A	2	R	<20	<20	<20	>50	>50	>50	>50	>50
TV1.29	C	ND	S	51	<20	45	>50	>50	>50	>50	3.38
CNE15	BC	ND	R	<20	21	37	>50	>50	>50	>50	>50
ZM55.28a	C	2	R	25	<20	40	>50	>50	>50	>50	>50
3589.V1.C4	AC	ND	R	<20	<20	59	>50	>50	>50	>50	>50
3873.V1.C24	C	2	R	<20	<20	73	>50	>50	>50	>50	5.32*
Q842.d12	A	2	R	29	39	53	>50	>50	>50	>50	>50
89.6.DG	B	2	S	<20	<20	90	>50	>50	>50	0.019	>50
BB539.2B13	A	ND	R	<20	<20	31	>50	>50	>50	>50	>50
RW020.2	A	2	R	42	23	53	>50	>50	>50	>50	34*
Ce1176.A3	C	2	R	34	<20	52	>50	>50	>50	>50	>50
BR07.DG	B	ND	S	<20	<20	42	>50	41.3	>50	0.541	>50
3301.V1.C24	AC	2	R	<20	<20	68	>50	>50	>50	>50	>50
CNE57	B	ND	S	80	34	227	49.3	>50	>50	1.01	>50
HO86.8	B	1B/2	S	<20	<20	59	>50	>50	>50	9.52	>50
MB201.A1	A	ND	R	28	<20	119	>50	>50	>50	>50	>50
ZM233.6	C	2	R	36	<20	51	>50	>50	>50	>50	35.8*
3365.v2.c20	A	2	S	<20	<20	29	>50	>50	>50	>50	0.689
CNE40	BC	2	S	76	73	171	0.062	>50	0.242	0.38	0.008
BB201.B42	A	ND	R	42	24	173	>50	>50	>50	>50	>50
Bal.01	B	ND	S	34	<20	100	>50	>50	>50	0.095	0.645
CH119.10	BC	2	R	23	<20	100	>50	>50	>50	>50	>50
MW965.26	C	1A	S	392	501	560	0.247	>50	>50	0.078	0.004
MI369.A5	A	ND	R	49	<20	200	>50	>50	>50	>50	>50
398-F1_F6_20	A	ND	R	159	31	436	>50	>50	>50	>50	>50
QH209.14M.A2	A	ND	R	<20	<20	54	>50	>50	>50	>50	>50
96ZM651.02	C	2	R	44	20	369	>50	>50	>50	>50	>50
CH117.4	BC	2	R	57	<20	311	>50	>50	>50	>50	>50
HXB2.DG	B	1B/2	S	28	<20	295	2.28	0.594	0.167	0.011	>50
ZM135.10a	C	2	R	<20	<20	<20	>50	>50	>50	>50	>50
ZM176.66	C	ND	R	<20	<20	64	>50	>50	>50	>50	>50
MB539.2B7	A	ND	R	<20	<20	37	>50	>50	>50	>50	>50
0921.V2.C14	C	2	R	<20	<20	<20	>50	>50	>50	>50	>50
CNE21	BC	2	R	25	<20	38	>50	>50	>50	>50	>50
BR025.9	C	1B/2	S	<20	<20	48	>50	>50	>50	>50	0.619
M02138	AE	ND	R	39	47	34	>50	>50	>50	>50	>50
242-14	AG	1B/2	R	40	25	31	>50	>50	>50	>50	15.4*
263-8	AG	2	R	31	53	44	>50	>50	>50	>50	>50
T257-31	AG	2/3	R	30	37	24	>50	>50	>50	>50	>50
6101.10	B	2	R	<20	<20	<20	>50	>50	>50	>50	>50
AC10.29	B	2	R	69	31	147	>50	>50	>50	>50	>50
BaL.26	B	1B	S	<20	<20	<20	>50	>50	10.9	0.066	0.855
BX08.16	B	1B	S	43	51	45	1.86	24.7	9.73	0.062	1.97
REJO.67	B	2	R	<20	<20	31	>50	>50	>50	>50	15.8*
001428-2.42	C	2	R	22	<20	<20	>50	>50	>50	>50	>50
25711-2.4	C	1B/2	R	<20	<20	<20	>50	>50	>50	>50	>50
6631.V3.C10	C	3	R	<20	<20	<20	>50	>50	>50	>50	>50
6838.V1.C35	C	2	R	40	<20	<20	>50	>50	>50	>50	>50
CNE53	C	2	R	<20	<20	<20	>50	>50	>50	>50	>50
DU123.06	C	2	R	<20	<20	205	>50	>50	>50	>50	15.5*
DU156.12	C	2	R	21	<20	<20	>50	>50	>50	>50	>50
SO18.18	C	ND	R	<20	<20	<20	>50	>50	>50	>50	>50
ZM106.9	C	2	R	54	43	114	>50	>50	>50	>50	>50
ZM249.1	C	2	R	<20	<20	22	>50	>50	>50	>50	>50
BJOX010000.06.2	AE	2	R	216	115	301	>50	>50	>50	>50	>50
C1080.c3	AE	2	R	<20	<20	<20	>50	>50	>50	>50	>50
C2101.c1	AE	2	R	<20	<20	34	>50	>50	>50	>50	>50
T278-50	AG	2/3	R	49	<20	23	>50	>50	>50	>50	>50
CAP45.G3	C	2	R	<20	<20	196	>50	>50	>50	>50	>50
SIVmac251.30.SG3	Non-HIV	N/A	N/A	<20	<20	49	>50	>50	>50	>50	>50
				**CGP701-1**	**CGP701-2**	**CGP701-5**					
**Total # Viruses**				60	60	60					
**Total # neutralized**				24	12	29					
**% neutralized**				40	20	48					
**Median ID50**				40	36	59					
**Geometric Mean**				45	42	77					

ID50
≥100
<100 and positive
Non-neutralizing

IC50 (µg/ml)
<0.01
0.01-0.1
0.1-1
1-10
10-50
>50
