# Supplementary material for: Consistent elicitation of cross-clade HIV-neutralizing responses achieved in guinea pigs after fusion peptide priming by repetitive envelope trimer boosting
Source: PLoS One. 2019 Apr 17;14(4):e0215163. doi: 10.1371/journal.pone.0215163 (PMC6469787; doi:10.1371/journal.pone.0215163)
Supplement: S5 Table — All samples were assessed for neutralization against non-HIV viruses, SIVmac251 and SVA-MLV, and ID50 values are listed. As can be seen, sporadic neutralization of non-HIV-1 viruses was observed. Based on these results, we considered neutralization titers positive for HIV-1 when ID50 ≥30 for CGP701-1 through CGP701-4 or when ID50 ≥50 for CGP701-5. We note that despite some non-specific neutralization by CGP701-5 sera, the neutralization fingerprint of this sera clustered most closely with the vaccine-elicited FP-directed antibodies (Fig 6), providing strong evidence that the observed neutralization by CGP701-5 was FP directed and not a background artifact. (PDF) [file pone.0215163.s005.pdf]

S5 Table. Non-HIV virus neutralization assessments. All samples were assessed for neutralization against non-HIV viruses, SIVmac251 and SVA-MLV, and ID_{50} values are listed. As can be seen, sporadic neutralization of non-HIV-1 viruses was observed. Based on these results, we considered neutralization titers positive for HIV-1 when $ID_{50} \geq 30$ for CGP701-1 through CGP701-4 or when $ID_{50} \geq 50$ for CGP701-5. We note that despite some non-specific neutralization by CGP701-5 sera, the neutralization fingerprint of this sera clustered most closely with the vaccine-elicited FP-directed antibodies (Fig 6), providing strong evidence that the observed neutralization by CGP701-5 was FP directed and not a background artifact.

	SIVmac251.30.SG3			SVA-MLV.SG3		
	week28	week36	week56	week28	week36	week56
CGP701-1	39	<20	<20	143	<20	<20
CGP701-2	<20	<20	<20	<20	<20	<20
CGP701-3	<20	<20	<20	<20	<20	<20
CGP701-4	<20	<20	<20	<20	<20	<20
CGP701-5	40	<20	49	49	<20	53
