# Supplementary material for: Consistent elicitation of cross-clade HIV-neutralizing responses achieved in guinea pigs after fusion peptide priming by repetitive envelope trimer boosting
Source: PLoS One. 2019 Apr 17;14(4):e0215163. doi: 10.1371/journal.pone.0215163 (PMC6469787; doi:10.1371/journal.pone.0215163)
Supplement: S6 Table — Sufficient sera from animals CGP701-1, -2, and -5 were available to assess FP competition for two Tier 2/R viruses from the 60-strain panel (S4 Table). Percentage reduction of neutralization was calculated by comparing the percentage neutralization in the presence of soluble FP9 peptide with that in the presence of media. Control assays were performed side-by-side using a Flag peptide. The assays were repeated two to three times, with representative data of one set of repeats reported. The assay results confirmed the reliability of the neutralization assays. Soluble FP could compete substantially, >30%, the neutralization by all three sera, except for virus 263–8 by CGP701-5 serum with 18% reduction in the presence of FP. The CGP701-5 serum had an ID50 of 44 against 263–8, below the cutoff titer of 50 for this serum (S5 Table). (PDF) [file pone.0215163.s006.pdf]

S6 Table. Fusion peptide competition of neutralization of two Tier 2 viruses. Sufficient sera from animals CGP701-1, -2, and -5 were available to assess FP competition for two Tier 2/R viruses from the 60-strain panel (S4 Table). Percentage reduction of neutralization was calculated by comparing the percentage neutralization in the presence of soluble FP9 peptide with that in the presence of media. Control assays were performed side-by-side using a Flag peptide. The assays were repeated two to three times, with representative data of one set of repeats reported. The assay results confirmed the reliability of the neutralization assays. Soluble FP could compete substantially, >30%, the neutralization by all three sera, except for virus 263-8 by CGP701-5 serum with 18% reduction in the presence of FP. The CGP701-5 serum had an ID_{50} of 44 against 263-8, below the cutoff titer of 50 for this serum (S4 and S5 Tables).

Animal ID	Virus	Clade	Control Media	FP_v1 peptide		Control Flag Peptide	
			% Neut	% Neut	Reduction of Neut	% Neut	Reduction of Neut
CGP701-1	263-8	AG	64.7	43.3	33%	64.3	1%
	ZM106.9	C	68.7	46.4	32%	71.7	-4%
CGP701-2	263-8	AG	58.2	31.7	46%	61.7	-0.1
CGP701-5	263-8	AG	77.2	63.3	18%	78.5	-2%
	ZM106.9	C	71.7	16.5	77%	74.9	-5%
