# Supplementary material for: Consistent elicitation of cross-clade HIV-neutralizing responses achieved in guinea pigs after fusion peptide priming by repetitive envelope trimer boosting
Source: PLoS One. 2019 Apr 17;14(4):e0215163. doi: 10.1371/journal.pone.0215163 (PMC6469787; doi:10.1371/journal.pone.0215163)
Supplement: S1 Fig — Guinea pig sera at week 56 post immunization were diluted at indicated reciprocal dilution starting from 1:20. Percentage neutralization was calculated by comparing with blank controls without serum. ID50 values were determined at 50% neutralization (dashed lines) by nonlinear regression curve fitting as detailed in the Method section. (PDF) [file pone.0215163.s007.pdf]

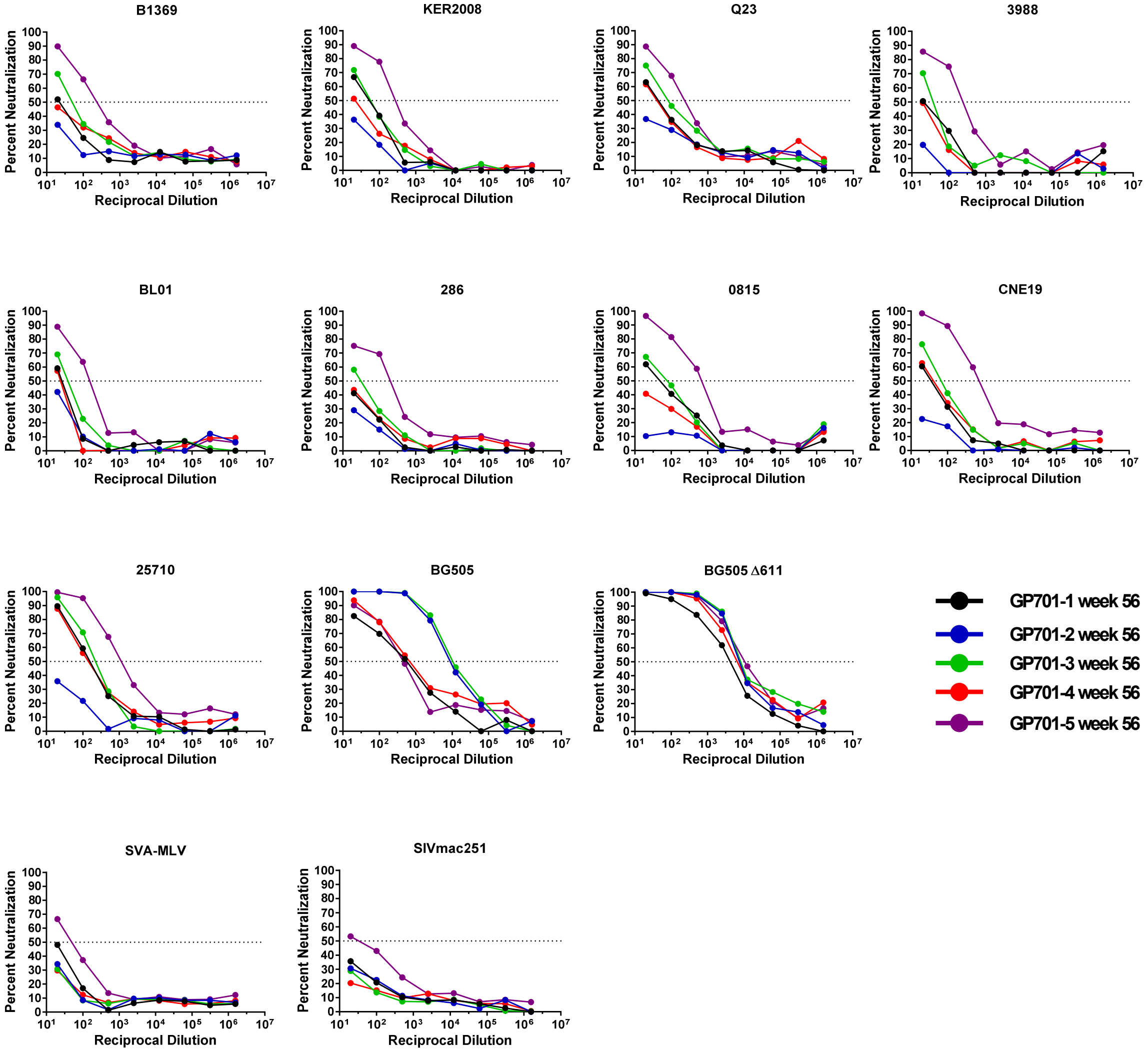

S1 Fig. Representative neutralization curves of guinea pig sera against HIV-1 and control viruses. Guinea pig sera at week 56 post immunization were diluted at indicated reciprocal dilution starting from 1:20. Percentage neutralization was calculated by comparing with blank controls without serum. Each data point is a single measurement. ID50 values were determined at 50% neutralization (dashed lines) by nonlinear regression curve fitting as detailed in the Method section.
