# Supplementary material for: Consistent elicitation of cross-clade HIV-neutralizing responses achieved in guinea pigs after fusion peptide priming by repetitive envelope trimer boosting
Source: PLoS One. 2019 Apr 17;14(4):e0215163. doi: 10.1371/journal.pone.0215163 (PMC6469787; doi:10.1371/journal.pone.0215163)
Supplement: S2 Fig — A. Serum neutralization breadth of glycan 241-containing and glycan 241-missing viruses for animals CGP701-1 to CGP701-5 at different time points. B. Differences in neutralization breadth for glycan 241-containing and glycan 241-missing viruses from different time points. Strains containing glycan 241: T278, 6644, 6405, A03349M1, ADA, C4118, TH023, BI369, KER2008, Q23, 3988, BL01, 286, 0815, and CNE19. Strains not containing glycan 241: BG505, CNE56, 25710, and 0077. (PDF) [file pone.0215163.s008.pdf]

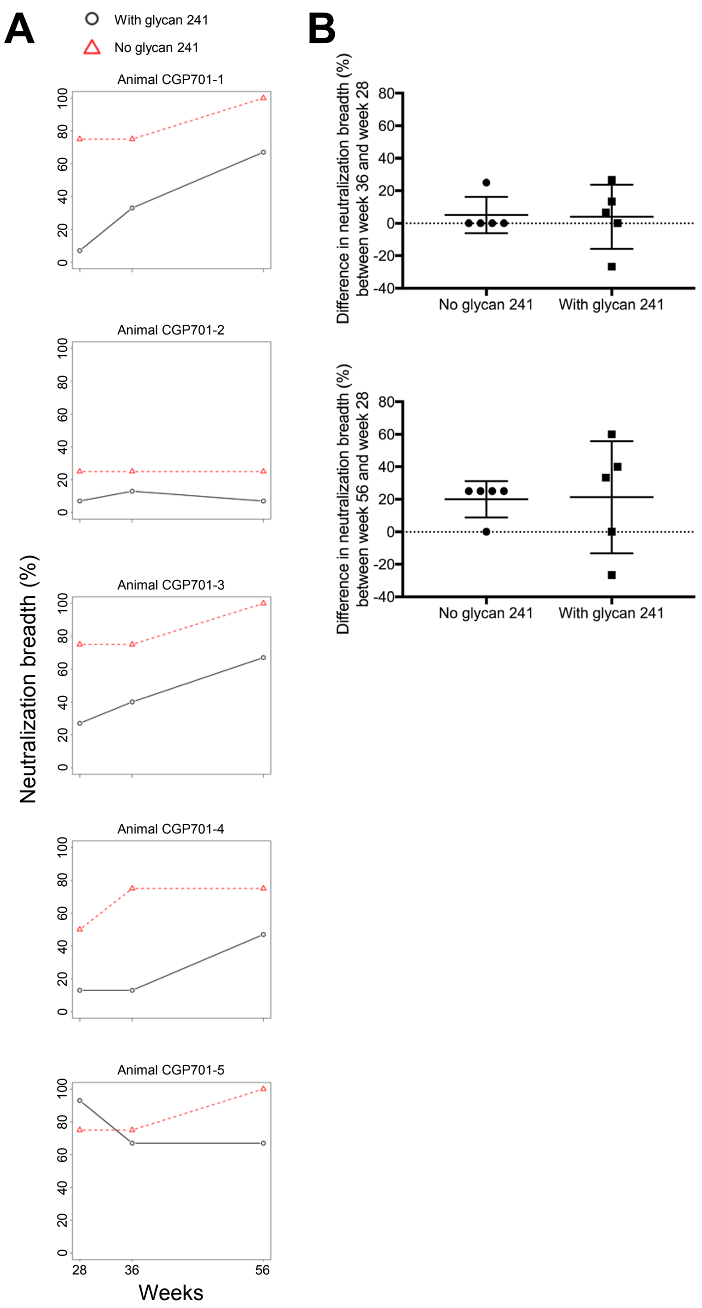

S2 Fig. Development of neutralization breadth upon CH505 trimer boost for glycan 241-containing and glycan 241-missing viruses. A. Serum neutralization breadth of glycan 241-containing and glycan 241-missing viruses for animals CGP701-1 to CGP701-5 at different time points. **B**. Differences in neutralization breadth for glycan 241-containing and glycan 241-missing viruses from different time points. Strains containing glycan 241: T278, 6644, 6405, A03349M1, ADA, C4118, TH023, BI369, KER2008, Q23, 3988, BL01, 286, 0815, and CNE19. Strains not containing glycan 241: BG505, CNE56, 25710, and 0077.
